# Simultaneous Determination of Six Chromones in Saposhnikoviae Radix via Quantitative Analysis of Multicomponents by Single Marker

**DOI:** 10.1155/2020/7867046

**Published:** 2020-04-14

**Authors:** Jinhua Zhang, Luxiao Chen, Junna Qiu, Yu Zhang, Lu Wang, Lijuan Jiang, Yanyan Jiang, Bin Liu

**Affiliations:** College of Traditional Chinese Medicine, Beijing University of Chinese Medicine, Beijing 102488, China

## Abstract

A method, quantitative analysis of multicomponents by single marker (QAMS), was established and fully verified based on high-performance liquid chromatography (HPLC) for simultaneous determination of six chromone indicators of Saposhnikoviae Radix (SR). In the present study, cimifugin (C), 5-O-methylvisamminol (V), hamaudol (H), and their corresponding glycosides, prim-O-glucosylcimifugin (GC), 4′-O-*β*-D-glucosyl-5-O-methylvisamminol (GV), and sec-O-glucosylhamaudol (GH), were selected as bioactive constituents and indicators for the quality evaluation of SR. GV was chosen as the unique reference standard, and relative correction factors (RCF) between GV and the other five chromones were calculated. The feasibility of QAMS for the analysis of chromones was investigated by comparing with the traditional external standard method (ESM). Furthermore, the method was proven to have accuracy (96.98%–102.50%), repeatability (RSD <3%), stability (RSD <3%), precision (RSD <3%), and desirable linearity (*R*^2^ ≧0.9999). Subsequently, 55 batches of commercial SR from different regions were determined by QAMS, and their contents were analyzed by principal component analysis (PCA), correlation analysis, and hierarchical cluster analysis (HCA), respectively. Based on the results, a more refined quality standard of commercial SR was proposed: SR was qualified when the total contents of six chromones were greater than 3 mg·g^−1^. Furthermore, SR could initially be regarded as a superior medicine when it satisfied both conditions at the same time: the total content of GC, C, GV, V, GH, and H was greater than 8 mg·g^−1^, and the proportion of the total content of C, V, and H was greater than 10%. This study demonstrated that the quality of SR could be successfully evaluated by the developed QAMS method; meanwhile, valuable information was provided for improving the quality standard of SR.

## 1. Introduction

Saposhnikoviae Radix (SR), known as “FangFeng” in China, is derived from the dried root of the plant *Saposhnikovia divaricate* (Turcz.) Schischk, belonging to the family Umbelliferae. This herb is pungent in flavour, sweet, and lukewarm, and enters the bladder, liver, and spleen meridian. According to the theory of Traditional Chinese Medicine (TCM), SR has significant effects on dispelling wind to relieve exogenous syndrome, removing dampness to kill pain, and stopping spasms [1]. As an important ingredient in many traditional Chinese prescriptions such as Yu-Ping-Feng-San, Fang-Feng-Tong-Sheng pill, and Tong-Xie-Yao-Fang [2, 3], SR has great application value. Furthermore, many pharmacological studies have indicated that a number of curative effects, including antipyretic, analgesic, anti-inflammatory, antibacterial, antitumor, antiallergic, and antioxidation, are existed in SR [4–8].

SR has several components: chromones, polysaccharides, coumarins, volatile oils, and other components [9–12]. It is worth mentioning that chromones are the most representative components in SR. On the one hand, there are extremely abundant content [13]. On the other hand, it was closely related to the pharmacological efficacy of SR, for example, anti-inflammatory, analgesic, and antioxidation [14–16]. With the development of SR market, increasing importance has been given to the quality control of SR. According to the record of Chinese Pharmacopoeia (2015 edition), prim-O-glucosylcimifugin (GC) and 4′-O-*β*-D-glucosyl-5-O-methylvisamminol (GV) were selected as quality-control indicators. However, each Chinese herbal medicine is an integrated complex with diversity of active components, contributing to particular efficacy through synergy and mutual effect based on the theory of TCM [17]. Thus, using a few components to control the quality is insufficient for the complex botanical products and traditional Chinese medicines. In line with the importance status of the chromones mentioned above, multiple representative chromone components could be selected as indicators to evaluate the quality of SR. According to phytochemical studies, GV, GC, sec-O-glucosylhamaudol (GH), cimifugin (C), 5-O-methylvisamminol (V), and hamaudol (H) are widely existing in SR [18, 19], and their pharmacological effects are significant and most related to the efficacy of SR [20, 21]. Therefore, when G, GC, V, GV, H, and GH are selected as new quality-control indicators, the chemical characterization, medicinal function, and inner quality of SR could be represented comprehensively. Some studies have shown that the methods of refluxing and ultrasonic were always used for the extraction of chromones [22, 23]. Considering the polarity and solubility of the six chromone indicators, the heating refluxing method could be applied for sample preparation, and the methanol could be used as the extraction solvent based on an optimization of sample preparation process in this work.

In many studies, the contents of multicomponents are usually determined by the external standard method (ESM). In this method, the reference standards are necessary that we need to spend more and more time and economic cost to separate and purify [24]. As an alternative method, quantitative analysis of multicomponents by single marker (QAMS) is only requiring a single reference standard to simultaneously determine the contents of multicomponents, which is more effective and appropriate for the quality control (QC) [25]. When some reference standards are unstable, low in abundance, or hard to extract from the plant, QAMS could not only reduce the cost but also reduce the difficulty in preparation [26, 27]. Besides, this method could improve the practicability of QC and expand the application for herbal or botanical products effectively. In QAMS, the content of internal standard could be obtained directly by HPLC and the other components could be calculated by using multiple conversion factors. Hence, the relative correction factor (RCF) is a critical parameter in the content computational formula about analytes. As the result of molar absorptivity of different analytes are often different, RFC plays a role of calibration when a single reference is used to determine multicomponents [28, 29]. In ESM, the concentration of analyte (*C*_*k*_) can be calculated by the ratio of the peak areas of analytes in sample solution (*A*_*k*_) to the peak area of its corresponding standard solution in a unit concentration (*A*_*s*_/*C*_*s*_), as shown in the following equation:

(1) $$\begin{array}{c} C_{k}=\frac{A_{k}}{A_{s}/C_{s}}. \end{array}$$

In QAMS, *C*_*k*_ should be calibrated by RCF of each analyte (*f*_*k*_) based on the calculation of ESM. The formula is as follows:

(2) $$\begin{array}{c} C_{k}=\frac{A_{k}}{A_{s}/C_{s}}\times f_{k}. \end{array}$$

Importantly, the value of *f*_*k*_ is calculated by the ratio of peak areas in a unit concentration of standard substance (*A*_*s*_/*C*_*s*_) to analyte (*A*_*k*_/*C*_*k*_) as follows:

(3) $$\begin{array}{c} f_{k}\frac{A_{s}/C_{s}}{A_{k}/C_{k}}. \end{array}$$

It is worth mentioning that the final value of RCF is usually the average value of multiple RCFs by a series of determining under different concentration levels of internal referring substance [30]. Since six control components, i.e., C, GC, V, GV, H, and GH, are used, ESM will cause high costs and complicated operations. Therefore, the QAMS method is used to compute the contents of six chromones, and GV is used as the internal standard for its strong representative nature, high stability, high content, and significant pharmacological activities [31, 32].

In the present study, a new substitute method named QAMS was applied for simultaneous determination of six chromones in SR. As the reference substance, the content of GV was determined by HPLC, and the contents of C, GC, V, H, and GH were calculated with RCF based on the intrinsic function and the proportional relationship between GV and these five chromones. The feasibility could be verified by comparing the results with the ESM, and this method was validated in terms of linearity, accuracy, precision (instruction precision and intermediate precision), and stability, referring to some reliable references [33]. Subsequently, 55 batches of commercial SR were determined and a more comprehensive and reliable quality evaluation standard of SR was preliminary inferred by principle component analysis (PCA), correlation analysis, and hierarchical cluster analysis (HCA), respectively.

## 2. Materials and Methods

### 2.1. Apparatus and Chromatographic Analysis

Analyses were primarily performed by using a Waters HPLC System (Waters Crop, Milford, MA, USA) equipped with a 1525 binary pump solvent management system, 2998 PDA detector, 2707 automatic sampling device, and Breeze 2 workstation. Two additional different HPLC instruments were used: One was high performance liquid chromatography (Waters Crop, Milford, MA, USA) equipped with 1525 binary pump solvent management system, 2489 UV detector, and Breeze 2 workstation. Another was a Waters Alliance e2695-2998 HPLC system (Empower workstation, Waters Crop, Milford, MA, USA). HPLC separation was carried out on a CAPCELL PAK C_18_ column (4.6 mm × 150 mm, 5 *μ*m). Column temperature was set at 25°C, and inject volume was 10 *μ*L. The mobile phase consisted of methanol (A) and 0.3% formic acid aqueous solution (B). The gradient elution was programmed at a flow rate of 1.0 mL·min^−1^ as follows: 0–12 min, 32% A; 12–40 min, 32%–50% A; 40–50 min, 50%–70% A; 50–52 min, 70% A. The detection wavelength was set at 254 nm.

### 2.2. Chemicals and Materials

**Figure 1:**
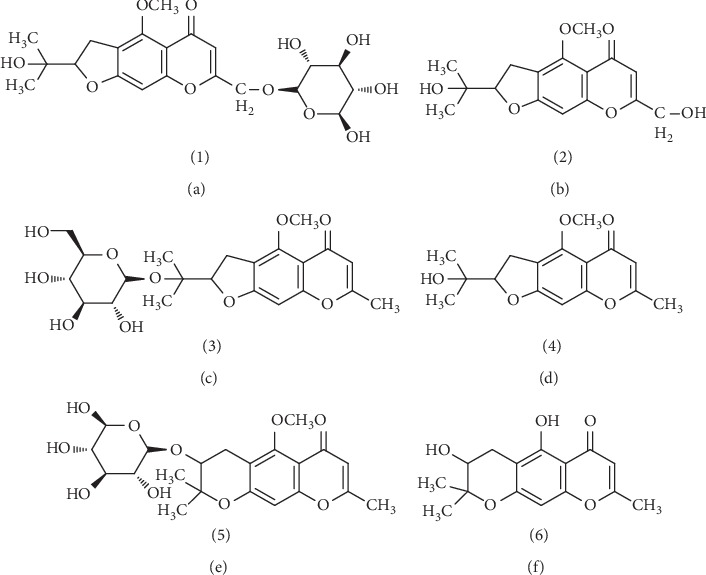
Chemical structures of prim-O-glucosylcimifugin (a), cimifugin (b), 5-O-methylvisammioside (c), 4′-O-D-glucosyl-5-O-methylvisamminol (d), sec-O-glucosylhamaudol (e), and hamaudol (f).

Fifty-five batches of SR were collected from different regions in China, which were identified by Professor Zhang Yuan from the Beijing University of Traditional Chinese Medicine and proved to be the dried root of *Saposhnikovia divaricate* (Turcz.) Schischk following the method described in Chinese Pharmacopoeia (2015 edition) [1]. GC, C, GH, and GV were obtained from Chengdu Mansite Biotechnology Co., Ltd. (Chengdu, China). The purity (≧98%) of these reference standards was assumed as provided by the suppliers. The other two compounds, V and H, were separated and purified in our lab and the purity was identified to be of not less than 98% (determined by HPLC). The structures were determined on the basis of UV, MS, and NMR data and confirmed by comparison with data from the literature. The chemical structures of all standards are shown in Figure 1.

Methanol, acetonitrile, and formic acid of HPLC grade were purchased from Thermo Fisher Scientific Inc. (Waltham, MA, USA), and other reagents (Beijing Chemical Industry Factory) were of analytical grade. HPLC grade water was prepared using a Pall Cascada IX system (Pall, USA). All other reagents were of analytical grade.

### 2.3. Preparation of Mixed Standard Solutions

Substances of C, GC, H, GH, V, and GV were weighed precisely and dissolved into methanol to prepare the mixed stock solutions of reference standards with the concentrations of 0.1220 mg·ml^−1^, 0.2096 mg·ml^−1^, 0.0369 mg·ml^−1^, 0.1636 mg·ml^−1^, 0.0808 mg·ml^−1^, and 0.3340 mg·ml^−1^, respectively. Then, a series of concentrations of calibration standard solutions were produced by diluting the mixed stock solution (dilution factor = 1, 2, 5, 10, 20, 50, and 100) with the same methanol. The solutions were stored at 4°C in a refrigerator and filtered through a 0.45 *μ*m membrane filter before injection. All samples being injected into HPLC system were prepared right before analysis.

### 2.4. Preparation of Sample Solutions

An appropriate amount of the samples to be tested was crushed and passed through an 80-mesh screen. Next, 0.25 g of the sample powder was precisely weighed and placed in a stuffed flask with accurate addition of 10 ml of methanol, subjected to heating reflux for 120 min. It is worth noting that the stuffed flask was weighed before and after refluxing, then added the solvent to keep the weight equivalent at room temperature if required. By filtering through the 0.45 *μ*m filter and discarding the first 2 ml, the remaining filtrate was used as the test solution of samples.

### 2.5. Method Validation

#### 2.5.1. Specificity

The mixed reference standard solution and test solution were separately injected into HPLC under the optimized chromatographic conditions (Section 2.1).

#### 2.5.2. Linearity

The prepared stock standard solutions mentioned above (Section 2.3) with a series of appropriate concentration levels were used for HPLC based on the chromatographic conditions (Section 2.1), respectively. The limits of detection (LOD) and quantification (LOQ) were measured based on a signal-to-noise ratio (S/N) at about 3 and 10, respectively.

#### 2.5.3. Precision

To ensure the validity of this newly developed method, the tests of instrument precision and intermediate precision were performed. For instrument precision, test solutions prepared (Section 2.4) were examined by HPLC for six replicates within one day, and on the purpose of detecting intermediate precision, the prepared test solutions were injected into HPLC by different operators with different instruments on different dates.

#### 2.5.4. Stability

The same tested solutions, which were prepared (Section 2.4) and placed at room temperature, were injected into HPLC at different time points (0, 2, 4, 8, 12, and 24 h).

#### 2.5.5. Repeatability

Six parallel sample solutions with the same batch were prepared following the method (Section 2.4) individually and determined by HPLC according to the chromatographic conditions (Section 2.1).

#### 2.5.6. Accuracy

Six copies of the same batch of SR powder (0.125 g) with known content were weighed, respectively. Then, a certain amount of control standard solution was added into the samples according to the proportion of sample content to reference substance, about 1 : 1. Preparation and determination of six sample solutions were conducted in parallel referring to the method as described in Sections 2.1 and 2.4. Recoveries were computed.

### 2.6. QAMS Method

#### 2.6.1. Calculation of RCF

Seven concentration levels of mixed standard solution were prepared (Section 2.3) and injected into HPLC under the chromatographic conditions (Section 2.1), respectively. Besides, the chromatographic peak areas of each component were recorded.

#### 2.6.2. Durability Test of RCF

Three different instruments (as listed in Section 2.1), three kinds of chromatographic columns (Capcell Pak C_18_, Water SunFire C_18_, and Water Symmetry C_18_) (4.6 mm × 150, 5 *μ*m), different flow rates (0.9, 1.0, and 1.1 ml·min^−1^), and different column temperatures (25, 30, and 35°C) were used to investigated the influence of different conditions on RCF.

#### 2.6.3. Location of the Chromatographic Peak of Measured Component

For better authentication as well as convenience to quality control of the commercial SR, the chromatographic peak positions of GC, C, GV, V, GH, and H were investigated using different instruments and different columns.

#### 2.6.4. Comparison of the Results between QAMS and ESM

In order to assess and validate QAMS feasibility of multicompounds in SR, the contents of C, GC, V, GV, H, and GV were determined by ESM and QAMS in 15 batches, respectively. For ESM, the determination of the six chromones was carried out with six reference standards (GC, C, GV, V, GH, and H), whereas for QAMS, the results were based on the nature of the calculation of *f*_*x*_, the intrinsic function, and the proportional relation between the selected reference analyte and other analytes. The content of the selected internal substance (GV) was determined like ESM, and then the contents of the other five chromones were calculated in accordance with relative conversion factors between analytes and the internal substance [34].

### 2.7. Application and Data Analysis

The developed QAMS method was applied for the quantitative assessment of 55 batches of commercial SR from different regions. The contents of GC, C, GV, V, GH, and H were determined and then analyzed by PCA, correlation analysis and HCA, respectively. Meanwhile, the further analysis of three chromone glycosides (C, H, and V) was carried out to make clarification of their importance for the overall quality of SR. The figures presented were developed by exploration of the analysis function using SPSS 22.0 software package.

## 3. Results and Discussion

### 3.1. Method Validation

#### 3.1.1. Specificity

**Figure 2:**
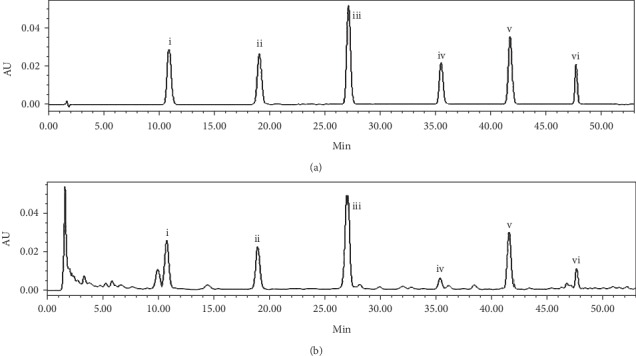
The chromatograms of the mixed reference standard solution (a) and test solution (b). The peaks represent (i) prim-(O)-glucosylcimifugin, (ii) cimifugin, (iii) 4′-(O)-*β*-D-glucosyl-5-(O)-methylvisammino, (iv) 5-(O)-methylvisamminol, (v) sec-(O)-glucosylhamaudol, and (vi) hamaudol.

As shown in Figure 2, the analytes had good separation because it has no interference in the corresponding position of the six components, and the target peaks of the test solution corresponded to the peaks of reference standard solution according to retention time in the chromatogram. So, it indicated that this method had specificity.

#### 3.1.2. Linearity

The standard curves of six reference substances were established by using the chromatographic peak area (*y*) as the vertical axis and the concentration of the reference solution (*x*) as abscissa, respectively. There was a good linearity as the result of all the correlation coefficients (*R*) which were not less than 0.999 over the concentration range. Furthermore, LOD and LOQ of six substances were also calculated as shown in Table 1.

#### 3.1.3. Precision

The areas of chromatographic peak and RSD of six compounds were recorded and calculated, respectively. In Table 2, the RSD results of six compounds for the instrument precision were in the range of 0.42%–0.75%. Apart from this, the RSDs which were calculated by intermediate precision were all lower than 3%. It indicated that this method has a good precision.

#### 3.1.4. Stability

The peak areas of GC, C, GV, V, GH, and H were recorded, and the values of RSD were 0.84%, 0.56%, 054%, 0.57%, 0.34%, and 0.69%, respectively. Hence, the sample solution was stable at room temperature within 24 h (Table 2).

#### 3.1.5. Repeatability

The results showed that average mass fractions of GC, C, GV, V, GH, and H were 1.478, 0.808, 2.510, 0.173, 1.053, and 0.151 mg·g^−1^, respectively. The RSD of corresponding average mass fraction was 1.03%, 0.73%, 0.49%, 0.80%, 0.96%, and 1.35% which proved that this quantitative method was of good repeatability.

#### 3.1.6. Accuracy

There was favorable accuracy because the average recovery rates of six marker compounds were varied in the range of 96.98%–102.5%. Meanwhile, RSD values of recovery rates for each compound were lower than 3% totally.

### 3.2. QAMS Method

#### 3.2.1. Calculation of RCF

GV was selected as the internal standard, and the values of RCF (*f*_*x*_) for other five indicators were computed in different concentrations according to the equation (3) mentioned above. The average RCF of each compounds was shown as follows: *f*_GC_ = 1.047 (RSD% = 2.32), *f*_C_ = 0.6489 (RSD% = 0.24), *f*_v_ = 0.5909 (RSD% = 0.30), *f*_GH_ = 0.7223 (RSD% = 0.68), and *f*_H_ = 0.7223 (RSD% = 0.28).

#### 3.2.2. Durability Test of RCF

The RCF of five chromones in different conditions (instruments, chromatographic columns, flow rates, and column temperatures) were obtained, and the RSDs were all less than 5%, which could clearly demonstrate that the RCF calculated by the proposed method has good durability and system suitability for routine testing.

#### 3.2.3. Location of the Chromatographic Peaks of Measured Components

The chromatographic peak position was identified by the relative retention time (RRT), which was calculated according to the following equation:

(4) $$\begin{array}{c} \Delta t_{\text{Rks}}=t_{\text{Rk}}-t_{\text{Rs}}, \end{array}$$

where *t*_Rk_ is the retention time of measured components, *t*_RS_ is the retention time of internal reference, and △*t*_Rks_ is the difference of retention time in both. Among them, the chromatographic peak position of GV was explicitly identified and designated as the reference peak in the SR samples. RRT was measured between GV and the other five components by different instruments and columns at the same time, and their RSDs were all less than 5%. It indicated that the calculation of RRT was stable and could be used for identifying the chromatographic peaks location of measured components.

#### 3.2.4. Comparison of the Results between QAMS and ESM

The contents of 15 batches of commercial SR were determined by the QAMS method and ESM. The results are shown in Table 3. Relative error (RE) was built between the two component variables to examine the deviations between QAMS and ESM. By comparing two sets of contents of five components between QAMS and ESM, respectively, their content variations were found to be within the range of 5%. It met the requirement of Chinese Pharmacopoeia. To assess the consistency of the results, correlation analysis was used to evaluate the similarity between the QAMS method and ESM. Correlation coefficient value is a commonly used parameter in the similarity evaluation. The larger the values, the higher the similarity of the target sample will be. When they are equal to 1, the targets are identical. In this work, the data, as shown in Table 4, were above 0.900, which indicated that there were no significant differences between the QAMS and ESM, and the identified RCF and parameters of chromatographic peak location for measured chromones of SR were reliable. In conclusion, QAMS can be applied in the determination of six chromones.

### 3.3. Application and Data Analysis

#### 3.3.1. Sample Analysis and Characteristics of Six Chromone Compounds in 55 Batches of Commercial SR

In 55 batches of commercial SR from different regions, the contents of six chromones, GC, C, GV, V, GH, and H, were determined by QAMS. Results are listed in Table 4. It was observed that the maximum total content of six compounds was 11.22 mg·g^−1^ in no. S53, while the minimum total content of those was 3.047 mg·g^−1^ in no. S18. Such a wide concentration variance of these 55 batches of commercial SR may be attributed to a variety of factors, including plant sources, genetic variation, and geography differences. To further verify the relationships among the samples and evaluate the variation of six compounds, PCA, coefficient analysis, and HCA were performed using the SPSS 22.0 software (IBM, USA).

#### 3.3.2. PCA and Correlation Analysis

**Figure 3:**
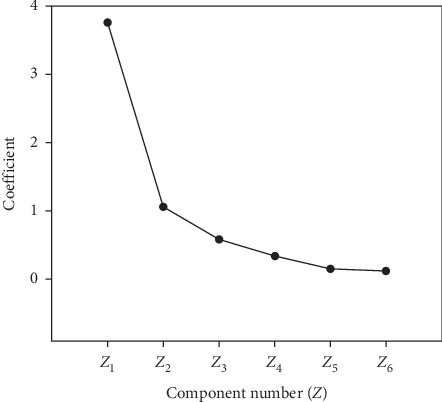
The coefficient of principal components.

**Figure 4:**
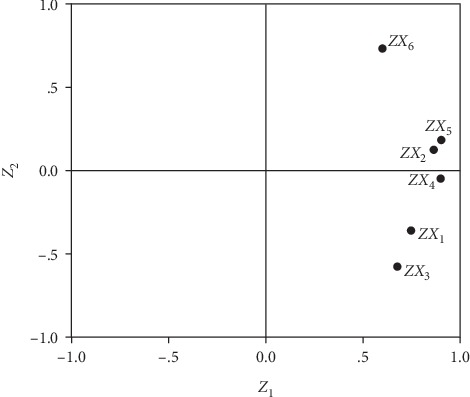
The coefficient between the content of 6 chromone compounds (*X*_1_, *X*_2_, *X*_3_, *X*_4_, *X*_5_, and *X*_6_) and *Z*_1_ and *Z*_2_.

The contents of GC (*X*_1_), C (*X*_2_), GV (*X*_3_), V (*X*_4_), GH (*X*_5_), and H (*X*_6_) of 55 batches of commercial samples were subjected to PCA. The results are shown in Figure 3. A two-component PCA model was established accounting for the accumulated variation of 80.260%, where the first principal component (*Z*_1_) was 62.614% and the second (*Z*_2_) was 17.646%. According to the component score coefficient matrix (Table 5; Figure 4), every coefficient between *Z*_1_ and six indicators was significant, which could indicate that *Z*_1_ represented the total content of six components. That is to say, each component was indispensable for the quality evaluation of SR. Furthermore, *Z*_2_ was mainly reflecting *X*_6_ as the result of their coefficient which was the largest (0.733). The comprehensive score (*Z*) for each batch sample could be obtained as follows:

(5) $$\begin{array}{c} Z=0.62614Z_{1}+0.17646Z_{2},\\ Z_{1}=0.749X_{1}+0.866X_{2}+0.679X_{3}+0.900X_{4}+0903X_{5}+0.599X_{6},\\ Z_{2}=-0.365X_{1}+0.122X_{2}-0.580X_{3}-0.049X_{4}+0.184X_{5}+0.733X_{6}. \end{array}$$

The details of 55 batche samples are shown in Table 6. Through correlation analysis of results from every batch sample, a good correlation between *Z* and the total content of six components could be included in accordance with the correlation coefficient which was 0.875. Thus, it was feasible to evaluate the quality of SR comprehensively if the total content of six components was used as indicator. Meanwhile, we preliminary set that the total content of six chromones should not be less than 3 mg·g^−1^ for qualified SR based on the above determined content range (3.047–11.22 mg·g^−1^).

#### 3.3.3. HCA

**Figure 5:**
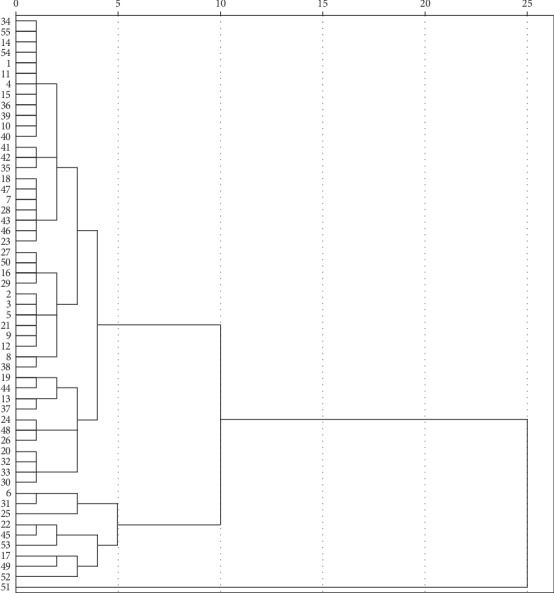
The cluster analysis tree of 55 batches of commercial SR (the indicator is the total content of six chromone compounds).

The HCA was applied to analyze the concentrations of GC, G, GV, V, GH, and H. The result indicated that 54 batches of commercial SRs (the 51st batch of SR was self-contained, not considered) were divided into two categories as shown in Figure 5. On the one hand, Cluster 1 consisted of 45 batch samples (34, 55, 14, 54, 1, 11, 4, 15, 36, 39, 10, 40, 41, 42, 35, 18, 47, 7, 28, 43, 46, 23, 27, 50, 16, 29, 2, 3, 5, 21, 9, 12, 8, 38, 19, 44, 13, 37, 24, 48, 26, 20, 32, 33, 30), in which their total content of six components was not more than 8 mg·g^−1^. On the other hand, the remaining 9 batch samples (6, 31, 25, 22, 45, 53, 17, 49, 52) were classified for second category, which were greater than 8 mg·g^−1^ of the indicator. Contacting with the results of Section 3.1.1, it could strongly prove that taking the total content of GC, G, GV, V, GH, and H as an indicator can reasonably, comprehensively, and objectively evaluate the quality of SR.

**Figure 6:**
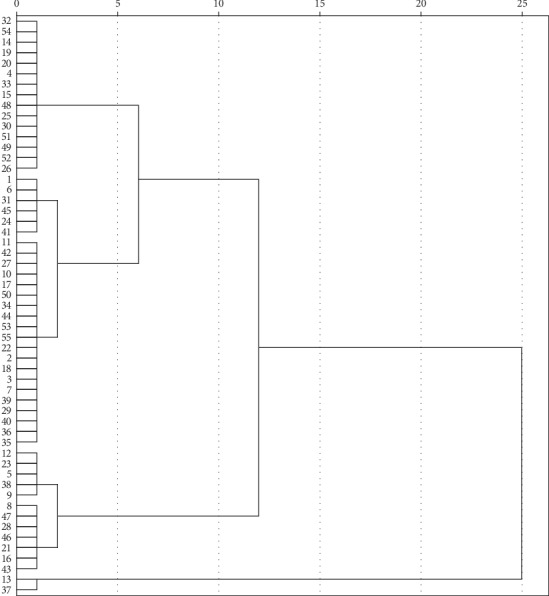
The cluster analysis tree of 55 batches of commercial SR (the indicator is the ratio of the total content of three chromone aglycones to the total content of six chromone compounds).

For the quality evaluation of SR, the chromone aglycone should not be ignored because this plays an important role in the pharmacological activity of SR. In addition, as shown in Table 5, the correlation coefficients of C, V, and H were significant (0.866, 0.900, and 0.0599), respectively. Using the ratio of the total content of C, V, and H to the total content of six chromones as an indicator, 53 batches of commercial SRs were divided into two categories by HCA (the 13th and 37th batches were self-contained, not considered). The details are shown in Table 7 and Figure 6. Cluster 1 included 41 batches (32, 54, 14, 19, 20, 4, 33, 15, 48, 25, 30, 51, 49, 52, 26, 1, 6, 31, 45, 24, 41, 11, 42, 27, 10, 17, 50, 34, 44, 53, 55, 22, 2, 18, 3, 7, 39, 29, 40, 36, 35), in which the ratio was greater than 10%. Meanwhile, the remaining 12 batch samples (12, 23, 5, 38, 9, 8, 47, 28, 46, 21, 16, 43) were attributed to Cluster 2, where the ratio was less than 10%. It was indicated that these three chromone aglycones were so important that they could be used as quality indicators for the quality evaluation of SR.

Last but not least, based on the results of the twice cluster analysis, samples of the Cluster 2 (6, 31, 25, 22, 45, 53, 17, 49, 52) in the first HCA were all contained to the Cluster 1 (32, 54, 14, 19, 20, 4, 33, 15, 48, 25, 30, 51, 49, 52, 26, 1, 6, 31, 45, 24, 41, 11, 42, 27, 10, 17, 50, 34, 44, 53, 55, 22, 2, 18, 3, 7, 39, 29, 40, 36, 35) in the second HCA. Thus, we could initially infer a conclusion that the SR could be regarded as a superior medicine when the total concentration of GC, C, GV, V, GH, and H was greater than 8 mg·g^−1^. Meanwhile, the proportion of aglycons (C, V, and H) was greater than 10%. In this study, 9 batch samples (6, 31, 25, 22, 45, 53, 17, 49, 52) were the superior medicine with the six chromone content which was greater than 8 mg·g^−1^, and meanwhile, the total content of C, V, and H was greater than 0.8 mg·g^−1^.

### 3.4. Optimization of Sample Preparation

Optimization of extraction methods, solvents, solvent volume, and extraction time were investigated by single-factor test to obtain the best extraction efficiency. The results revealed that extraction efficiency of refluxing was more efficient than ultrasonic extraction for analytes, so the remaining experiment was carried out by refluxing, and methanol was chosen as the best solvent by comparing with various solvents including methanol, 70% methanol, and 50% methanol. In addition, the extraction volume (5, 10, and 15 ml) and the extraction times (60, 120, and 240 min) were tested subsequently. Consequently, the optimal sample preparation parameter was refluxing with 10 mL methanol for 120 min as for 0.25 g powder of SR.

## 4. Conclusion

A method named QAMS was established to evaluate the quality of SR based on routine HPLC apparatus. In this method, GV was chosen as the internal standard to determine the RCF between GV and other five chromones (GC, C, V, GH, and H) of SR. QAMS was accurate and feasible for the quality evaluation according to the results of method validation, and no significant difference existed in the content results obtained by QAMS and ESM. Using this developed method, 55 batches of commercial SR from different regions were determined, and the results were analyzed by PCA, correlation analysis, and HCA, respectively. It could include that chromones played an important role for the quality of SR; meanwhile, the total content of GC, C, GV, V, GH, and H was used as the evaluation indicator that was comprehensive, objective, and reliable. Other than this, the SR could be regarded as qualified medicine if the total content of six chromones was not less than 3 mg·g^−1^. Moreover, the importance of chromone aglycones for the quality evaluation of SR was further demonstrated. It could initially infer that it was a superior SR medicine if the total content of GC, C, GV, V, GH and H was greater than 8 mg·g^−1^; meanwhile, the proportion of C, V, and H was greater than 10%.

All in all, these results provided useful information for the development of commercial SR, and the quality of SR from different origins or different purchase locations was confusing and unstable. Therefore, a compulsive processing standard for SR should be established and standardized. Last but not least, the abovementioned series of analyses will play a positive role in the improvement of the quality evaluation system of SR.

## Figures and Tables

**Table 1:** Regression equations, correlation coefficients, linearity ranges, limits of detection, and limits of quantification for six indicators.

Indicator	Regression equation^1^	*R*	Linearity range (*μ*g·ml^−1^)	LOD (*μ*g·ml^−1^)	LOQ (*μ*g·ml^−1^)
Prim-*O*-glucosylcimifugin	*y* = 1815300*x* + 8028	0.9999	2.10–209.60	0.29	0.96
Cimifugin	*y* = 2928940*x* + 7329	0.9999	1.22–122.00	0.28	0.92
4′-*O*-*β*-D-glucosyl-5-*O*-methylvisammino	*y* = 1894482*x* + 14466	0.9999	3.34–334.00	0.28	0.94
5-*O*-methylvisamminol	*y* = 3222987*x* + 4879	0.9999	0.81–80.80	0.18	0.61
Sec-*O*-glucosylhamaudol	*y* = 2660022*x* + 6702	0.9999	1.64–163.60	0.22	0.73
Hamaudol	*y* = 4418335*x* + 1757	0.9999	0.37–36.90	0.09	0.31

^1^In the regression equation *y* = a*x* + *b*, where *y* refers to the peak area and *x* refers to the concentration of the indicator (*μ*g·mL^−1^).

**Table 2:** Instrument precision, intermediate precision, stability, repeatability, and recovery of six analytes.

Indicator component	Instrument precision (*n* = 6)	Intermediate precision (*n* = 6)	Stability (*n* = 6)	Repeatability (*n* = 6)	Accuracy (*n* = 6)	
	RSD (%)	RSD (%)	RSD (%)	RSD (%)	Mean recovery (%)	RSD (%)
Prim-*O*-glucosylcimifugin	0.75	2.4	0.84	1.03	99.87	1.66
Cimifugin	0.56	1.69	0.56	0.73	97.34	0.77
4′-*O*-*β*-D-glucosyl-5-*O*-methylvisamminol	0.43	1.23	0.54	0.49	96.98	1.31
5-*O*-methylvisamminol	0.42	0.36	0.57	0.8	102.5	1.03
Sec-*O*-glucosylhamaudol	0.37	1.06	0.34	0.96	98.13	0.71
Hamaudol	0.67	1.45	0.69	1.35	99.48	0.68

**Table 3:** Comparison of contents by QAMS and ESM for CV as internal standard (mg·g^−1^).

No.	GV	GC			C			V			GH			H		
	ESM	ESM	QAMS	Relative error (%)	ESM	QAMS	Relative error (%)	ESM	QAMS	Relative error (%)	ESM	QAMS	Relative error (%)	ESM	QAMS	Relative error (%)
1	2.896	4.295	4.289	−0.14	1.048	1.047	−0.10	0.09829	0.09793	−0.37	1.069	1.070	0.09	0.06459	0.06460	0.02
2	0.8574	2.646	2.642	−0.15	0.3916	0.3914	−0.05	0.04527	0.04511	−0.35	0.2362	0.2364	0.08	0.1061	0.1061	0.00
3	1.889	3.783	3.778	−0.13	0.7479	0.7475	−0.05	0.06943	0.06918	−0.36	0.9545	0.9553	0.08	0.1507	0.1507	0.00
4	2.480	2.885	2.881	−0.14	0.2100	0.2098	−0.10	0.05922	0.05901	−0.35	0.2845	0.2847	0.07	0.020008	0.02009	0.05
5	2.848	3.510	3.505	−0.14	0.9254	0.9248	−0.06	0.08080	0.08051	−0.36	0.7045	0.7051	0.09	0.06158	0.06159	0.02
6	1.832	2.019	2.016	−0.15	0.6268	0.6264	−0.06	0.05237	0.05219	−0.34	0.2341	0.2343	0.09	0.02235	0.02236	0.04
7	1.961	2.241	2.239	−0.09	0.5400	0.5396	−0.07	0.05959	0.05937	−0.37	0.2508	0.2510	0.08	0.07276	0.07278	0.03
8	2.062	2.249	2.246	−0.13	0.2459	0.2458	−0.04	0.06527	0.06503	−0.37	0.2155	0.2157	0.09	0.0256	0.02565	0.00
9	1.390	4.122	4.123	0.02	0.3810	0.3809	−0.03	0.0398	0.03918	−0.51	0.4580	0.4593	0.28	0.08772	0.08785	0.15
10	3.961	2.466	2.466	0.00	1.469	1.469	0.00	0.2263	0.2251	−0.53	1.264	1.267	0.24	0.3047	0.3051	0.13
11	2.004	3.054	3.054	0.00	1.491	1.491	0.00	0.1301	0.1294	−0.54	0.7997	0.8018	0.26	0.1760	0.1763	0.17
12	2.519	2.363	2.363	0.00	1.029	1.030	0.10	0.08996	0.08953	−0.48	0.6982	0.6997	0.21	0.06253	0.06263	0.16
13	2.848	4.417	4.416	−0.02	1.754	1.756	0.11	0.1763	0.1754	−0.51	0.9507	0.9528	0.22	0.1461	0.1463	0.14
14	2.233	4.983	4.983	0.00	0.799	0.800	0.09	0.07737	0.07700	−0.48	0.8065	0.8082	0.21	0.07475	0.07487	0.16
15	4.038	3.972	3.973	0.03	1.239	1.238	−0.08	0.1380	0.1372	−0.58	0.6367	0.6384	0.27	0.05454	0.05462	0.15

Correlation coefficient		0.999996894			0.999998781			0.999996565			0.99999876			0.999999518		

**Table 4:** Content of six chromone compounds in 55 batches of commercial SR.

No.	Compound content (mg·g^−1^)						
	GC	C	GV	V	GH	H	Total
1	1.317	0.4275	1.261	0.02920	0.2946	0.03354	3.363
2	2.418	0.4976	2.382	0.04779	0.3659	0.03554	5.747
3	2.651	0.5458	2.698	0.06159	0.3781	0.02972	6.364
4	1.337	0.6792	1.654	0.04434	0.2859	0.06364	4.064
5	2.689	0.2197	2.876	0.03020	0.3112	0.01964	6.146
6	4.573	1.107	1.955	0.03020	0.5887	0.08134	8.335
7	1.729	0.4001	1.835	0.03092	0.1675	0.02509	4.188
8	2.564	0.3944	3.718	0.04341	0.2723	0.02125	7.013
9	2.430	0.1014	2.255	0.03667	0.2496	0.01529	5.088
10	2.258	0.6732	2.216	0.00000	0.3463	0.05838	5.552
11	1.304	0.344	1.449	0.03023	0.3543	0.06303	3.545
12	2.452	0.1243	2.755	0.03687	0.1534	0.05423	5.576
13	1.346	0.9504	1.465	0.06466	0.3278	0.11580	4.270
14	1.777	0.5765	1.127	0.02988	0.2396	0.08358	3.834
15	1.786	0.6144	1.364	0.03312	0.1714	0.05189	4.021
16	2.896	0.422	2.682	0.06387	0.5827	0.09963	6.746
17	4.69	1.02	2.551	0.10200	1.1490	0.15070	9.663
18	1.043	0.2811	1.573	0.02553	0.1242	0.00000	3.047
19	1.717	0.6368	1.962	0.08296	0.2502	0.15210	4.801
20	2.28	0.983	2.533	0.09785	0.3417	0.07771	6.313
21	2.729	0.3448	2.464	0.04038	0.2646	0.02275	5.866
22	4.2	1.147	3.442	0.14280	0.6112	0.06122	9.604
23	2.139	0.1695	1.929	0.00000	0.3146	0.01560	4.568
24	2.744	0.8955	2.324	0.08142	0.7392	0.1081	6.892
25	4.45	1.843	2.509	0.09471	0.6873	0.07324	9.657
26	2.817	1.401	2.608	0.09266	0.9524	0.07638	7.947
27	2.368	0.5529	2.202	0.05162	0.3305	0.07492	5.580
28	1.487	0.1982	1.880	0.03231	0.1906	0.01684	3.805
29	3.599	0.6820	2.030	0.04863	0.5045	0.06149	6.926
30	2.116	1.4090	3.498	0.11720	0.3130	0.04773	7.501
31	4.237	1.2030	1.818	0.03805	0.7812	0.06076	8.138
32	2.077	0.9935	2.653	0.08411	0.3187	0.07907	6.205
33	2.221	1.1080	2.931	0.10130	0.2647	0.06694	6.693
34	1.863	0.3630	0.941	0.03012	0.3419	0.07997	3.619
35	1.818	0.3075	1.096	0.00000	0.3717	0.12460	3.718
36	2.134	0.3686	1.076	0.00000	0.1803	0.06089	3.820
37	2.173	1.3010	1.232	0.04985	0.5113	0.12210	5.389
38	3.773	0.3078	3.220	0.06403	0.3651	0.02726	7.757
39	2.234	0.4056	1.289	0.00000	0.1985	0.03652	4.164
40	2.168	0.4823	1.776	0.00000	0.2772	0.05842	4.762
41	1.490	0.4874	1.240	0.00000	0.3820	0.12980	3.729
42	1.708	0.3880	1.587	0.00000	0.3404	0.13020	4.154
43	1.834	0.2949	2.261	0.03667	0.1705	0.02114	4.618
44	2.199	0.5615	2.569	0.06130	0.2619	0.13020	5.783
45	3.667	1.3310	3.572	0.12560	0.8210	0.08447	9.601
46	1.985	0.1671	1.610	0.04060	0.1129	0.03844	3.954
47	1.765	0.2047	1.508	0.03583	0.1699	0.00000	3.683
48	2.363	1.0300	2.519	0.08953	0.6997	0.06263	6.764
49	3.670	1.3180	1.900	0.11270	0.8467	0.18620	8.034
50	2.447	0.5516	1.778	0.07714	0.4362	0.07921	5.369
51	2.466	1.4690	3.961	0.22510	1.2670	0.30510	9.693
52	4.416	1.7560	2.848	0.17540	0.9528	0.14630	10.300
53	5.136	1.2960	3.769	0.17010	0.7804	0.07168	11.220
54	1.901	0.5797	0.953	0.03516	0.2994	0.10780	3.876
55	1.825	0.3481	0.867	0.03686	0.3172	0.09194	3.486

**Table 5:** The component score coefficient matrix.

	Principal component	
	*Z*_1_	*Z*_2_
*X*_1_	0.749	−0.365
*X*_2_	0.866	0.122
*X*_3_	0.679	−0.580
*X*_4_	0.900	−0.049
*X*_5_	0.903	0.184
*X*_6_	0.599	0.733

**Table 6:** Comprehensive evaluation of 55 batches of commercial SR.

No.	*Z*
1	−2.2088
2	−0.8149
3	−0.3972
4	−1.2788
5	−1.3328
6	−1.4037
7	−2.187
8	−0.7398
9	−1.9465
10	−1.1080
11	−1.8149
12	−1.5472
13	−0.1805
14	−1.5233
15	−1.7940
16	0.6962
17	4.3433
18	−3.1265
19	0.1636
20	0.7331
21	−1.2827
22	3.0727
23	−2.3900
24	1.7230
25	3.4021
26	2.8143
27	−0.4977
28	−2.5341
29	0.3312
30	1.4697
31	1.6586
32	0.5130
33	0.7921
34	−1.6345
35	−1.5166
36	−2.3571
37	0.8094
38	−0.0475
39	−2.3745
40	−1.7203
41	−1.2931
42	−1.2774
43	−2.0675
44	0.0793
45	3.6328
46	−2.3500
47	−2.7293
48	1.3859
49	3.8177
50	−0.0636
51	7.7397
52	5.6501
53	4.5777
54	−1.1113
55	−1.5619

**Table 7:** Ratio of the total content of three chromone aglycones to the total content of six chromones.

No.	Ratio
1	14.58
2	10.11
3	10.01
4	19.37
5	4.39
6	14.62
7	10.89
8	6.55
9	3.01
10	13.18
11	12.34
12	3.86
13	26.49
14	18.00
15	17.39
16	8.68
17	13.17
18	10.06
19	18.16
20	18.35
21	6.95
22	14.07
23	4.05
24	15.74
25	20.82
26	19.76
27	12.18
28	6.50
29	11.44
30	20.98
31	16.00
32	18.64
33	19.07
34	13.07
35	11.62
36	11.24
37	27.33
38	5.14
39	10.62
40	11.36
41	16.55
42	12.48
43	7.64
44	13.02
45	16.05
46	6.23
47	6.53
48	17.48
49	20.13
50	13.19
51	20.62
52	20.18
53	13.70
54	18.64
55	13.68

## Data Availability

All chromatographic data used to support the findings of this study are available from the corresponding author upon request.
